# Multi-Angle Fusion-Based Safety Status Analysis of Construction Workers

**DOI:** 10.3390/ijerph182211815

**Published:** 2021-11-11

**Authors:** Hui Deng, Zhibin Ou, Yichuan Deng

**Affiliations:** 1 School of Civil Engineering and Transportation, South China University of Technology, Guangzhou 510641, China; hdeng@scut.edu.cn (H.D.); 201921007707@mail.scut.edu.cn (Z.O.); 2 State Key Laboratory of Subtropical Building Science, Guangzhou 510641, China

**Keywords:** worker detection, multiple cameras, trajectory estimation, safety analysis, intelligent management

## Abstract

Hazardous accidents often happen in construction sites and bring fatal consequences, and therefore safety management has been a certain dilemma to construction managers for long time. Although computer vision technology has been used on construction sites to identify construction workers and track their movement trajectories for safety management, the detection effect is often influenced by limited coverage of single cameras and occlusion. A multi-angle fusion method applying SURF feature algorithm is proposed to coalesce the information processed by improved GMM (Gaussian Mixed Model) and HOG + SVM (Histogram of Oriented Gradient and Support Vector Machines), identifying the obscured workers and achieving a better detection effect with larger coverage. Workers are tracked in real-time, with their movement trajectory estimated by utilizing Kalman filters and safety status analyzed to offer a prior warning signal. Experimental studies are conducted for validation of the proposed framework for workers’ detection and trajectories estimation, whose result indicates that the framework is able to detect workers and predict their movement trajectories for safety forewarning.

## 1. Introduction

Although there has been a reduction in fatal construction injuries, the construction industry still has the highest number of casualties of any other industry, which means the safety of the construction industry still needs attention to avoid fatal accidents [1]. Previous research on the application of computer vision technology to the detection and tracking of on-site workers has shown its feasibility for safety management on construction sites. However, when utilizing computer vision technology, the detection effect is unsatisfactory because of the limited coverage of single cameras and occlusion. Moreover, there is little study in this area of automatic system analyzing workers’ safety status. The problems mentioned above can be summarized as the following aspects:

(1) Is there a worker detection method that can adapt to the dynamic and ever-changing environment of the construction site?
(2) Is there a worker detection method that can fuse visual information from multiple angles to avoid occlusion problems?
(3) Is there a method with low computational cost for the management of safety of workers on the construction site?

To overcome the knowledge gaps, this paper proposes an improved method of worker detection with multi-angle information fusion and realizes the prediction of movement trajectory to determine their safety status and offer a prior warning signal, contributing to the on-site safety management. This paper focuses on the real-time detection of the safety status of on-site workers, proposing two improvements: (1) multi-angle detection of construction workers; (2) low demand for computational resources for detection of workers. Due to the changeable nature of construction sites, the arrangement of the detection system should be replicated easily without a laborious and tedious process of training a neural network. The framework for workers’ detection should be light and simple, and able to identify workers from multiple angles to solve the above problems. Experimental studies were carried out to verify the effectiveness of the presented framework.

At present, numerous techniques have been applied to the safety management of construction sites [2,3,4]. However, among these techniques, the manual method is low-efficient, high-cost, and subjective [5,6,7], and methods with wearable equipment need frequent charging and high application cost, affecting the efficiency of workers [8,9,10]. Overcoming the limitation of traditional methods, computer vision technology is considered to realize intelligent management on construction sites [11,12,13], especially for automatic recognition and monitoring of on-site workers [14,15]. Moreover, previous research indicates that on-site safety performance can be improved by computer vision technology (e.g., detection of safety equipment, motion analysis, and tracking of workers) [16,17]. With the advantages of computer vision technology, recognition and tracking of workers has become a trend in safety management, aiming to detect the workers’ unsafe behavior and status on construction sites.

Despite its benefits, there are still some limitations of existing studies on recognition and tracking of on-site workers. Weerasinghe and Ruwanpura [18] set up an AMOT (automatic multi-target tracking system) to monitor the on-site workers and equipment while analyzing both audio and video. Dong et al. [19] proposed a proactive struck-by risk detection method for workers proximal to the laydown area to enhance construction site safety, but the implementation of this method is dependent on tag-based tracking technology. Luo et al. [12] came up with a hierarchical statistical method for capturing and understanding workers’ high-level activities in far-field surveillance videos but creating data sets to train the TSNs is indispensable and expensive when new workers’ actions are introduced. Yang et al. [20] established a tracking algorithm based on the machine learning method to track multiple workers on construction sites using video cameras with poor recognition effect unfortunately. Brilakis et al. [21] presented a vision-based tracking framework to identify and track construction entities, providing 3D spatial coordinates of entities. Ren et al. [22] proposed project related entities tracking on construction sites by particle filtering to overcome the problem that the detection target is blocked, showing the importance of solving the problems caused by occlusion. Guo et al. [23] pointed out that existing location technologies can perform well only in relatively small areas due to their generally poor penetrating performance. After reviewing many relevant studies, Zhong et al. [24] argued that multiple cameras are needed to be placed on the site to fill up some knowledge gaps in the field of computer vision technology. Moreover, Park et al. [25] employed a method of transforming two-dimensional coordinates into three-dimensional coordinates by using the on-site camera system, realizing the tracking of construction resources. However, the processing of the video is not real-time. In another study, the location data of workers and construction equipment was collected and processed by visual detection technology, and relevant safety performance information was displayed to decision-makers in real time, aiming to achieve the safety management of on-site workers [26], but the information collected had not been processed automatically.

As can be seen, some studies still require miscellaneous conditions (e.g., the placement of tags) to recognize and track workers’ movement trajectories. Although attempts of using computer vision to detect and track workers in construction sites have been developed, the identification effect is still poor and several factors affect the accuracy of worker’s identification, especially occlusion. Therefore, multiple cameras are needed to be placed on the site, coalescing the information they gather to achieve wider coverage and improve the detection effect. What is more, the result of detection and tracking should be processed automatically in real time and the movement estimation is crucial for safety management [27,28]. In general, this paper intends to solve the following research problems:

(1) A background extraction method for solving the influence of illumination mutation.
(2) A multi-angle worker detection method for solving the occlusion problem.
(3) A method with low computational cost that can adapt to the construction site.

To address these limitations and implement an automatic process of site safety management, the improved GMM (Gaussian Mixed Model) and HOG + SVM (Histogram of Oriented Gradient and Support Vector Machines) framework are offered, enhancing the effect of workers’ detection. Meanwhile, the information fusion of workers’ detection result from multiple angles is realized by utilizing the SURF (Speeded Up Robust Features) feature algorithm, overcoming the limited coverage of single cameras and achieving a better detection effect. The effectiveness of the method mentioned above was tested on real site videos collected by two cameras. Kalman filter was applied to predict the movement trajectory of construction workers without any pre-positioned labels, after which the workers’ safety status can be determined automatically to offer a prior warning signal when they enter a danger zone. In general, this paper proposes an improved method of workers’ detection utilizing multi-angle fusion and establishes an automatic analysis system for the safety status of on-site workers. The framework in this paper does not require the installation of any sensors on workers, which makes the method available at most construction sites, especially the large-scale ones because of the larger coverage of multiple cameras. Moreover, this paper provides an idea to implement automatic safety management in construction sites, helping to manage the safety of construction sites and prevent accidents from happening.

## 2. Methodology Overview

**Figure 1:**
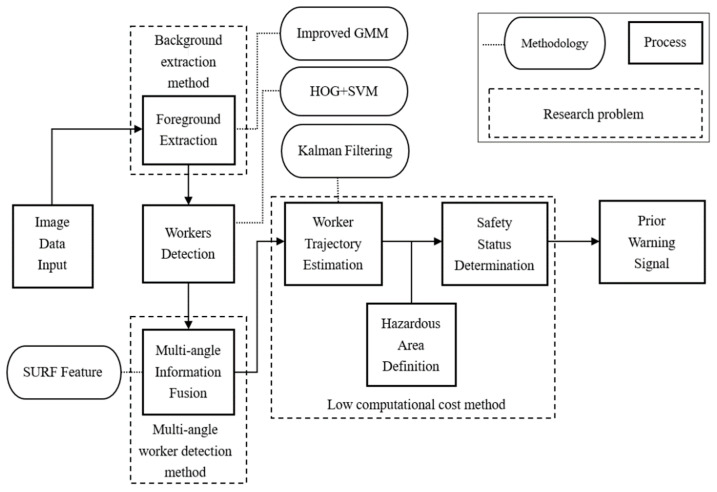
Overview of the framework.

This paper introduces an automated method for the detection of on-site workers from multiple angles and the prediction of movement trajectory. This section is organized as follows: (1) introduction of the methods and some improvement idea utilized in this paper, such as improved GMM, HOG feature and SVM, SURF feature, and Kalman filtering; (2) the improved GMM and HOG + SVM framework for the detection of construction workers; (3) the determination of the safety status of construction workers based on path prediction; (4) discussion, conclusion, insufficient and future expectation. The following content of this paper is shown in Figure 1.

### 2.1. Improved GMM

In this paper, the video background refers to the relatively stable picture content in the video, that is, the fixed scene with few changes, while the moving object in the video is considered as the foreground, the specific target to be detected. The purpose of background modeling is to segment the relatively stable part of the video frame from the moving target, achieving the dichotomy between the foreground and background of the video frame content.

**Figure 2:**
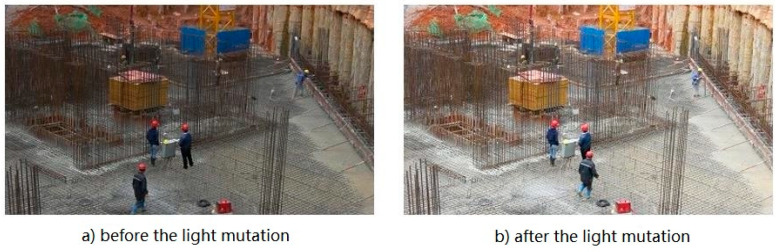
Light changes suddenly.

The GMM background modeling method is to establish the color distribution model of a single pixel in the video according to the statistical law of time. This method is suitable for accurate modeling of complex backgrounds such as light gradation and tree sway. However, in the actual application, the abrupt change of the scene (shown in Figure 2) may lead to the misjudgment of the algorithm. For consideration of this effect, a corresponding improvement to the GMM background modeling method is proposed in this paper.

When the overall brightness of the scene changes suddenly, the GMM background model cannot adapt to such changes. This mutation is mathematically represented as the change in the value of all pixels in image, and its effect is considered according to the average change of pixel values in the common background between adjacent video frames. For the video frames at time *t*, the pixel areas of each moving entity in the video frames at time *t* − 1, *t* − 2, and *t* − 3 are known. The centroid coordinates of these areas are used to represent the positions of construction workers, and each movement area needs to be marked with a different number. Therefore, according to the difference of centroid coordinates of the same digital marker region in the continuous video frame before time *t*, the velocity and acceleration of each moving target can be obtained, as shown in Formula (1).

(1) $$\{\begin{array}{c} v^{l}=S_{t-1}^{i}-S_{t-2}^{i}\\ a^{l}=S_{t-1}^{i}-2S_{t-2}^{i}+S_{t-3}^{i} \end{array}$$

In Formula (1), $S_{t-1}^{i}$ and $S_{t-2}^{i}$ represent the pixel coordinates of the centroid of the *i*th motion region, $S^{i}=\left(S_{x}^{i},S_{y}^{i}\right)$. $v^{l}$ represents the estimated velocity of the centroid of the *i*th motion region at time *t*, $v^{l}=\left(v_{x}^{l},v_{y}^{l}\right)$. $a^{l}$ represents the estimated acceleration of the centroid of the *i*th motion region at time *t*, $a^{l}=\left(a_{x}^{l},a_{y}^{l}\right)$. According to Formula (1), $v^{l}$ and $a^{l}$ are solved, and then the positions of each target *i* at time *t* can be predicted by Formula (2).

(2) $$S_{t}^{l}=S_{t-1}^{i}+v^{l}+\frac{1}{2}a^{l}$$

**Figure 3:**
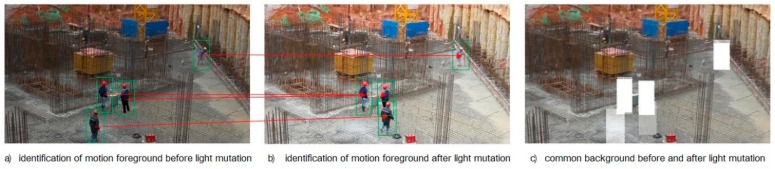
Common background of the first and second frames.

Taking $S_{t}^{l}$ as the centroid of a rectangle, the prediction region of each target at time *t* can be identified. If the boundary rectangle region of each target at time *t* − 1 and the predicted boundary rectangle region at time *t* are taken as the union set, then for time *t* − 1 and time *t*, the complementary set region of this union in the image is the common background component, as shown in Figure 3. The pixel bits corresponding to the region of the video frame at time *t* are subtracted from the pixel bits corresponding to the region of the video frame at time *t* − 1 to obtain the changes in the pixel values of the background region. The distribution of the change of background pixel value can be obtained by collecting the gray histogram of the change of each pixel value in the background region. If the overall brightness of the scene does not change suddenly, the mean value of the distribution theoretically approaches 0. If the overall brightness of the scene changes suddenly, the mean value of the distribution will shift greatly and the variance will be small.

Before applying the GMM background model for foreground extraction, the mean value of the distribution is first used to modify the mean value of the Gaussian distribution in the GMM model, and the modified formula is shown in Formula (3), in which $\mu_{j,t-1}^{i}$ and $\mu_{j,t-1}^{i}{}^{\prime }$, respectively, represent the mean Gaussian distribution before and after illumination change. $\bar{\mu_{t}}$ represents the average variation in background illumination brightness.

(3) $$\mu_{j,t-1}^{i}{}^{\prime }=\mu_{j,t-1}^{i}+\bar{\mu_{t}}$$

### 2.2. HOG Feature and SVM

HOG feature calculates and counts the gradient direction of each pixel in the local area of the image to establish the histogram, which is widely used for the pedestrian detection. To obtain the HOG feature of the image, the gradient direction of each pixel of the image is firstly calculated and divided the value range n of the gradient direction into a finite sub-interval, so that the direction can be discretized. Then, a designated detection window is set as a statistical region and divided into subranges to construct multiple histograms of direction gradient of the local region. According to the subordination relationship between the gradient orientation value and each orientation sub-interval, all the pixels within the cell unit are voted and counted for each discrete sub-interval to obtain the HOG feature of the cell unit. Finally, the HOG features of each cell unit are serialized into a vector to be the HOG features of the local region.

In the field of machine learning, SVM is a typical classifier and achieves classification by creating an optimal decision hyperplane that maximizes the distance from the samples closest to it. For the case of linear inseparability, the general principle is to map the samples from the original sample space to a higher dimensional space, so that the samples in this higher dimensional space become linearly separable. It has been proved mathematically that if the original space is finite dimensional, there must be a higher dimensional feature space that makes the sample linearly separable.

With the development of deep learning, most of the common target detection methods have been based on CNN, which needs a large training set with high training cost and is time-consuming [29,30]. In order to build a simple and fast target detection system, this paper adopts SVM based on machine learning to detect workers, which adapts to the limited and changeable field computing resources.

### 2.3. SURF Feature

The SURF feature is a robust method for detecting and describing local feature points in images, which takes specific pixel points in the image as the processed object. The SURF algorithm, along with its high efficiency, has a better matching effect than other matching algorithms in the case of obvious light changes, which are frequent on construction sites. Therefore, the SURF algorithm meets the requirements of the proposed lightweight framework [31]. The SURF feature utilizes the Hessian matrix to construct the determination of mutation points, in preparation for feature extraction. The specific form of Hessian matrix is a square matrix composed of the second partial derivative of a multivariate function and describes the local curvature of the function.

Since the image is a discrete binary function, the image pixel value in a small local area may also have mutations and the image in this small local area is often slippery. The specific form of Hessian matrix is Formula (4), which is a square matrix composed of the second partial derivative of a multivariate function and describes the local curvature of the function. The larger the determinant value of *H* matrix is, the larger the change value of local curvature of the corresponding point is, and the point with greater local curvature is more likely to be a corner point. Therefore, the discriminant for the feature point to be selected by using *H* matrix is Formula (5). $T_{H}$ is the threshold set for distinguishing feature points to be selected.

(4) $$H\left[I\left(X,Y\right)\right]=\left[\begin{array}{cc} \frac{\partial^{2}I}{\partial x^{2}} & \frac{\partial^{2}I}{\partial x\partial y}\\ \frac{\partial^{2}I}{\partial y\partial x} & \frac{\partial^{2}I}{\partial y^{2}} \end{array}\right]$$

(5) $$Det\left(H\right)=\frac{\partial^{2}I}{\partial x^{2}}\cdot \frac{\partial^{2}I}{\partial y^{2}}-{\left(\frac{\partial^{2}I}{\partial x\partial y}\right)}^{2}>T_{H}$$

The image is a discrete binary function, the image pixel value in a small local area may also have mutations (noise points, noise spots, etc.), so the image in this small local area is often slippery. The abrupt change of pixel value in such a small local area often interferes with the information provided by the image and affects the determination of the feature point to be selected (*Det(H)* of the noise point is large, but it is not the corner point). In order to improve the consistency with the mathematical model and the accuracy of the algorithm, before the numerical model analysis of the image, the image is often smooth processing (fuzzy processing), removing the image noise and noise spots and other interference information. The commonly used image smoothing method is applying Gaussian kernel to carry out convolution operation on the whole image, and its mathematical expression is shown in Formula (6).

(6) L(x,y;σ)=G(σ)×I(x,y)

For the image, the H-matrix after Gaussian smoothing is shown as Formula (7). Accordingly, the discriminant of h-matrix for feature point discrimination becomes Formula (8). In practical application, in order to use the integral graph to improve the operation rate, the SURF algorithm uses the Gaussian filter. To balance the error caused by using the box filter approximation, the term of the discriminant is multiplied by the coefficient 0.9, so the discriminant is changed to Formula (9).

(7) $$H\left(x,y;\sigma \right)=\left[\begin{array}{cc} L_{xx}\left(x,y;\sigma \right) & L_{xy}\left(x,y;\sigma \right)\\ L_{yx}\left(x,y;\sigma \right) & L_{yy}\left(x,y;\sigma \right) \end{array}\right]$$

(8) $$Det\left(H\right)=L_{xx}\cdot L_{yy}-{\left(L_{xy}\right)}^{2}>T_{H}$$

(9) $$Det\left(H\right)=L_{xx}\cdot L_{yy}-{\left(0.9\cdot L_{xy}\right)}^{2}>T_{H}$$

**Figure 4:**
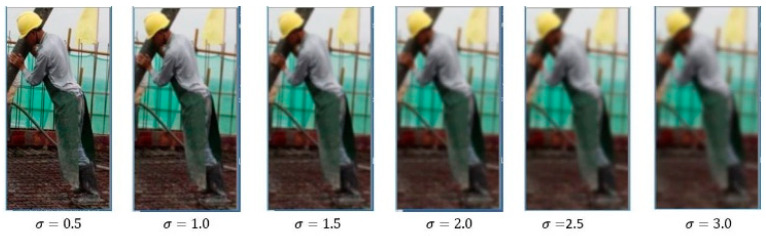
Effect of different blurring degrees.

In order to construct the scale invariance of feature points (after the image is scaled to different sizes, all pixel points in the same position can be determined as feature points), it is necessary to construct the scale space of the image. The image is scaled to different sizes at a certain scale, and the image pyramid formed by these scaled images set as scale space. Different sizes of the same image can be reversely seen as different blurring degrees of the image. The Gaussian smoothing is to blur the image and the blurring degree is controlled by σ. Different degrees can be constructed by adjusting the size of σ to obtain the scale space of the image, as shown in Figure 4.

The directional invariance of feature points refers to the fact that in photos of the same scene under different shooting conditions, the feature points have certain directional invariability in two different pictures. Harr small baud in the circular neighborhood of statistical feature points is utilized to construct directional invariance. In the circular neighborhood of the feature points, with the horizontal axis as the starting direction and the 0.2 radian size as the step size, the sum of the horizontal and vertical Harr wavelet features of all points in the π/3 radian sector subregion is calculated, respectively. Then the direction of the sector with the largest sum of the wavelet features was selected as the principal direction of the feature points. After constructing the principal direction of the feature points to be selected, the feature points should meet the same principal direction on different images, so that the direction of the feature points is invariant.

### 2.4. Kalman Filtering

The location prediction of construction workers refers to the prediction of workers’ movement trajectory by establishing the model of workers’ dynamic movement. Kalman filter is a commonly used method to predict dynamic systems. In linear random systems with interference, Kalman filter can be used to predict the future state of the system, and the error of the predicted value is smaller than that of the direct observation value. In order to apply Kalman filter, the linear stochastic system model of the target needs to be established in advance, that is, state modeling and measurement modeling. State modeling is to determine the state transfer equation, which is the state recursive function of time, while measurement modeling is to determine the measurement equation, which is the function of state. In this paper, the state transfer equation is a recursive linear equation set of worker coordinate positions with process noise interference, and the measurement equation is a linear equation set of worker coordinate positions with measurement noise interference.

After establishing the linear stochastic system, an arbitrary initial state is assumed for the system. The value of the state is obtained from the sensor and the state transfer equation is used to update the state of the system. Thus, the updated state variables and measurement equations are used to obtain the measurements of the target. Moreover, Kalman filtering reduces the errors generated by the noise of the system by combining all historical observations.

In order to apply Kalman filter, state modeling is needed to determine the state transition equation. In this paper, the Singer acceleration model is used to establish the motion state equation of construction workers [32,33]. The Singer model assumes that the target acceleration a(t) is a stationary first-order Markov process with zero mean value, thus obtaining the state of the linear time-invariant system as shown in Formula (10). It can be seen from Formula (10) that the parameter σ^2^ is the instantaneous change of acceleration and is treated as a random variable.

(10) $$\dot{a}=-\alpha a\left(t\right)+w\left(t\right)$$

In Formula (10), $\dot{a}$ represents the first derivative of the acceleration vector. α is the inverse of the maneuver time τ_m_ (10~20 s). a(t) and w(t) represent acceleration vector and zero mean Gaussian white noise vector, respectively. According to Formula (10), its discrete form can be obtained, namely Formula (11).

(11) $$a_{k+1}=\beta a_{k}+w_{k}^{a}$$

In Formula (11), $w_{k}^{a}$ is a sequence of zero mean white noise vectors with variance $\sigma^{2}\left(1-\beta^{2}\right)$. β is a constant. For $w_{k}^{a}$, $\beta =e^{-\alpha T}$. Assuming that the state variable is *x* ($x={\left[x,\dot{x},\ddot{x}\right]}^{T}={\left[x,v,a\right]}^{T}$), the state space of the continuous time singer model of Formula (10) is expressed as Formula (12), and the equivalent discrete form of Formula (11) is obtained as Formula (13). In Formula (13), *T* represents the unit time interval.

(12) $$\dot{x}\left(t\right)=\left[\begin{array}{ccc} 0 & 1 & 0\\ 0 & 0 & 1\\ 0 & 0 & -\alpha \end{array}\right]x\left(t\right)+\left[\begin{array}{c} 0\\ 0\\ 1 \end{array}\right]w\left(t\right)$$

(13) $$x_{k+1}=F_{a}x_{k}+w_{k}=\left[\begin{array}{ccc} 1 & T & \left(\alpha T-1+e^{-\alpha T}\right)/\alpha^{2}\\ 0 & 1 & \left(1-e^{-\alpha T}\right)/\alpha \\ 0 & 0 & e^{-\alpha T} \end{array}\right]x_{k}+w_{k}$$

The discrete time linear stochastic dynamic system is described in Formula (14). In Formula (14), *k* stands for timing indicators (k∈N). $x_{k}$ represents the system state vector at time *k* ($x_{k}\in R^{m}$). $z_{k}$ represents the direction finder for the system state at time *k* ($z_{k}\in R^{m}$). $F_{K},\;w_{k},\;\Gamma_{k},\;H_{K},\;v_{k}$ represent the system state transition matrix, process evolution noise, noise driven matrix, measurement matrix, and measurement noise, respectively. For the discrete-time linear stochastic dynamic system described by Formula (14), the one-step advance prediction for measurement is expressed as Formula (15), assuming that all random variables are Gauss. The corresponding one-step advance prediction error sequence is shown in Formula (16), which is called the new information sequence.

(14) $$\{\begin{array}{c} x_{k+1}=F_{K}x_{k}+\Gamma_{k}w_{k}\\ z_{k}=H_{K}x_{k}+v_{k}\;\;\;\; \end{array}$$

(15) $${\hat{z}}_{k|k-1}=E\left(z_{k}|Z^{k-1}\right);\;k\in N$$

(16) $${\hat{z}}_{k|k-1}=z_{k}-{\hat{z}}_{k|k-1};\;k\in N$$

If the random variable is non-Gauss, it can be estimated by the BLUE criterion (Best Linear Unbiased Estimate, the optimal linear unbiased estimation), and Formula (17) can be obtained. At this point, Formula (18) shows the corresponding prediction error sequence, which is called the pseudo-novelty sequence.

(17) $${\hat{z}}_{k|k-1}{}^{\prime }=E*\left(z_{k}|Z^{k-1}\right);\;k\in N$$

(18) $${\hat{z}}_{k|k-1}{}^{\prime }=z_{k}-{\hat{z}}_{k|k-1}{}^{\prime };\;k\in N$$

If the discrete-time linear stochastic dynamic system described by Equation (14) is assumed to be $w_{k}\sim N\left(0,Q_{k}\right)$ and $v_{k}\sim N\left(0,R_{k}\right)$ independent processes, independent of each other, and independent of the initial state $x_{0}\sim N\left({\bar{x}}_{0},P_{0}\right)$, then the basic Kalman filtering formulas of Formulas (19) to (24) are valid for any situation loss function. By taking Formula (13) as the state equation of the discrete time linear stochastic dynamic system described by Formula (17), the movement trajectory of construction workers can be predicted according to Formula (20), and the error of the predicted position is Formula (21).

(1) The initial condition.
  (19) $$\{\begin{array}{c} {\hat{x}}_{0|0}={\bar{x}}_{0}\\ {\hat{x}}_{0|0}=x_{0}-{\hat{x}}_{0|0}\\ cov\left({\hat{x}}_{0|0}\right)=P_{0} \end{array}$$
(2) The predicted value of one step in advance.
  (20) $${\hat{x}}_{|k-1}=E\left(x_{k}|Z^{k-1}\right)=F_{k-1}{\hat{x}}_{k-1|k-1}$$
(3) The covariance matrix of one-step predicted value error.
  (21) $$P_{k|k-1}=cov\left({\tilde{x}}_{k|k-1}\right)=F_{k-1}P_{k-1|k-1}F_{K-1}^{T}+\Gamma_{k-1}Q_{k-1}\Gamma_{K-1}^{T}$$
  In Formula (21), ${\tilde{x}}_{k|k-1}$ stands for prediction error. ${\tilde{x}}_{k|k-1}=x_{k}-{\hat{x}}_{k|k-1}$.
(4) The filter update value after the new measurement is obtained.
  (22) $${\hat{x}}_{k|k}=E\left(x_{k}|Z^{k}\right)={\hat{x}}_{k|k-1}+K_{K}\left(z_{k}-H_{k}{\hat{x}}_{k|k-1}\right)$$
(5) The corresponding filtering error covariance matrix of the filter update value.
  (23) $$P_{k|k}=cov\left({\tilde{x}}_{k|k}\right)=P_{k|k-1}-P_{k|k-1}H_{K}^{T}{\left(H_{k}P_{k|k-1}H_{K}^{T}+R_{k}\right)}^{-1}+H_{k}P_{k|k-1}$$
  In Formula (23), ${\tilde{x}}_{k|k}$ stands for the filtering error.$\;{\tilde{x}}_{k|k}=x_{k}-{\hat{x}}_{k|k}$.
(6) The Kalman gain matrix at time *k*.
  (24) $$K_{k}=P_{k|k-1}H_{k}^{T}{\left(H_{k}P_{k|k-1}H_{k}^{T}+R_{k}\right)}^{-1}$$

## 3. Multi-Angle On-Site Worker Detection

### 3.1. Foreground Extraction

#### 3.1.1. Motion Foreground Separation

**Figure 5:**
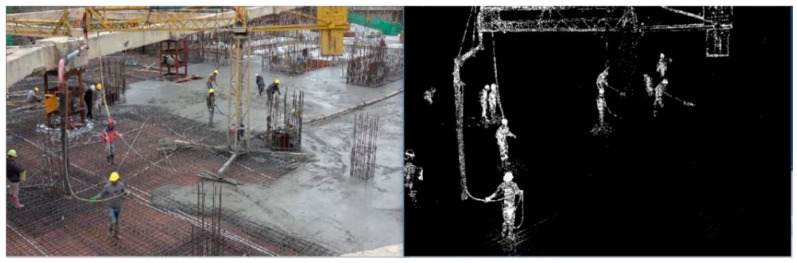
Moving object detection.

Before foreground detection, Gaussian mixture model is used to model each background pixel in the video frame, and then the background is trained. After the background is extracted through the model training, the detection of the frame foreground becomes checking whether the pixels of the video frame match the Gaussian model corresponding to the background. If it matches the Gaussian model, it is the background pixel. Otherwise, it is the foreground pixel, and the foreground is extracted from the pixel level. The effect of utilizing the GMM background modeling method to extract moving objects on the construction site is shown in Figure 5. After extracting all movable objects from the site, the paper will classify these moving objects to accurately distinguish construction workers from other moving objects.

#### 3.1.2. Image Noise Removal

**Figure 6:**
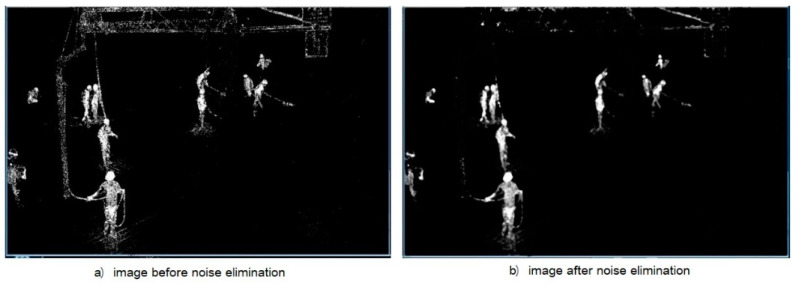
Noise elimination.

As can be seen from Figure 6, although the GMM background modeling method can identify the motion foreground, it also detects many noise points. Since the main information is not lost, the noise can be removed, which can effectively improve the detection effect of the target object. Commonly used in computer vision, the denoising method includes the maximum filtering, the minimum filtering, median filtering, Gaussian filtering, Gaussian bilateral filtering, and mean shift filtering. After test, the effect of the median filter is relatively optimal. Therefore, the median filter was chosen for noise elimination in this paper, and the effect after the elimination of noise is shown in Figure 6.

#### 3.1.3. Morphological Operation

**Figure 7:**
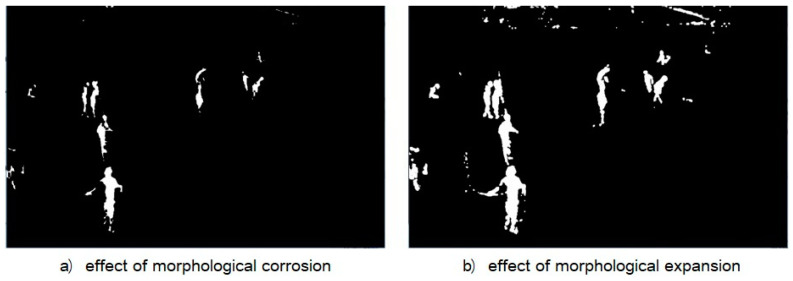
Morphological operation.

**Figure 8:**
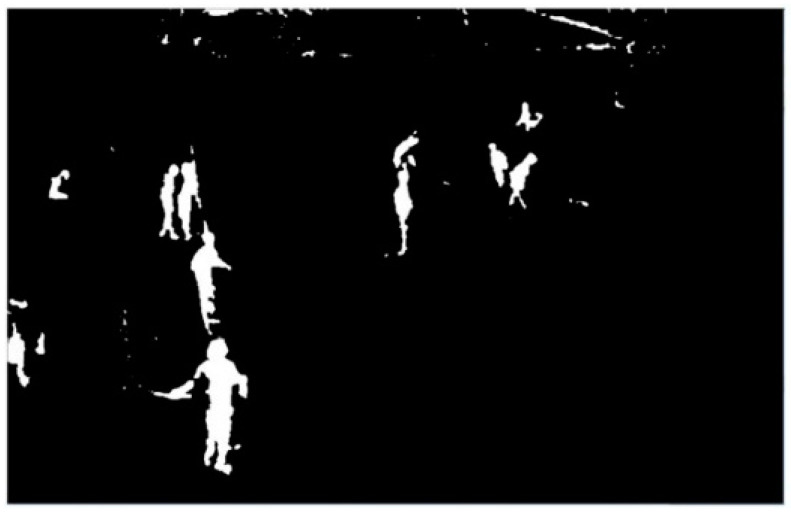
Open operation.

It can be seen from Figure 6b that there are still some unnecessary noises in the de-noised image, and there is no obvious separation between the moving foreground entities. Additionally, there is the case that the entities are connected to each other to be indistinct. In practice, the foreground segmentation results may also be empty in some moving entities and the solid contour region cannot be obtained. Morphological etching can eliminate small and meaningless noise spots in the image and reduce the edge thickness of the target to separate different entities (Figure 7a). In contrast to the morphological corrosion operation, the morphological expansion operation can enlarge the edge thickness of the target object, so that the small holes inside the foreground entity can be filled (Figure 7b). The operation of morphological expansion after morphological corrosion is called morphological open operation. Repeated open operation in Figure 7b can alleviate or eliminate the influence of the above problems such as noise spots and cavities, and the treatment effect is shown in Figure 8.

#### 3.1.4. Motion Foreground Marker

**Figure 9:**
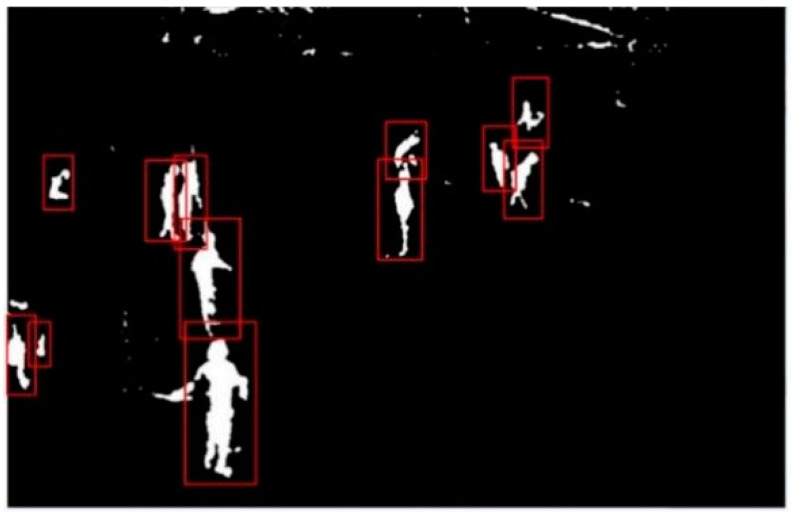
Windows of moving object identification.

After the previous processing, the moving solid contour in the motion foreground can be separated, and many unnecessary noise spots and non-target objects have been removed, so that the rectangular box can be used to identify the independent moving solid contour areas. By searching the connected region of the whole image, all the moving foreground entities can be obtained. In this paper, by limiting the height to width ratio of the identified target boundary box to 1.5~2 and the area limit to 1000 pixels, the detection of some non-target moving foreground entity contour can be ignored. The detection and identification results of moving entity contour are shown in Figure 9. The sub-image identified in the boundary box shows the moving solid contour area as white and the other areas as black. The binary sub-image can not only identify the position of the moving entity in the image, but also be used to eliminate the non-target redundant pixels in the corresponding range of the original input image.

### 3.2. On-Site Worker Detection

**Figure 10:**
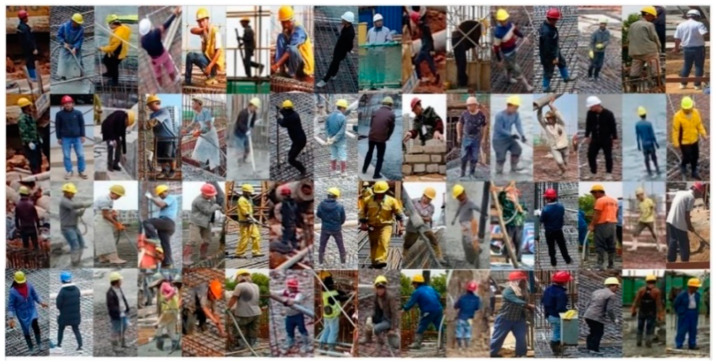
SVM training set.

In total, 1100 images of construction workers were collected by taking images on a construction site (shown in Figure 10). The image dataset covers workers of different genders, postures, and construction procedures. The HOG features of these images were extracted for training the classifier, to distinguish construction workers from other objects. When real-time video is input, the HOG feature of the moving object in the video is extracted, and the trained classifier can classify and discriminate the sub-images in the boundary box. In the OpenCV visual library, the HOG feature object is first created by using the class cv2.HOGDescriptor (winSize, blockSize, blockStride, cellSize, nbins), and then the HOG feature of each sample image can be calculated by its sub-function cv2.HOGDescriptor().compute().

Gaussian kernel, linear kernel, and polynomial kernel are separately selected to establish the mapping relationship between low-dimensional and high-dimensional spaces, and the SVM classifier is trained by combining HOG features of each training sample to classify the moving objects in the video. In the OpenCV visual library, SVM instances can be created by the class cv2.ml.SVM_create(). Data learning and training are performed by the member function train() of the SVM instance, and the training result data is saved by the member function of the SVM instance. To be classified, the training result data saved above is called by using cv2.ml.SVM_load(), and the member function predict() of SVM instance is used to classify the new data.

**Figure 11:**
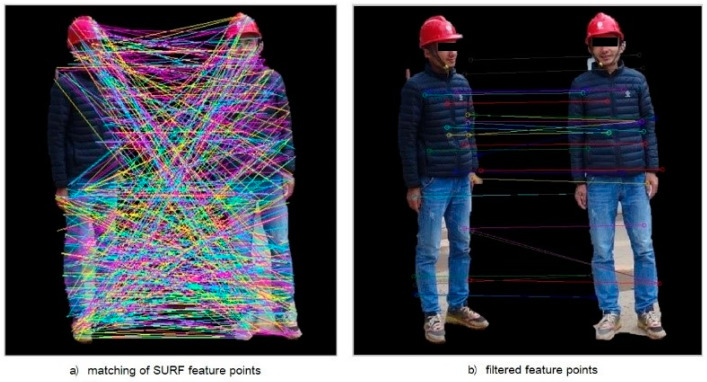
SURF feature point matching.

The background modeling method is used to extract the moving objects in the video, and then the sub-images of moving objects are detected and classified. The improved method can effectively avoid the classification misjudgment caused by the close distribution of local texture in the static image and improve the operation efficiency. As for the integration of the detection information from different angles under the condition of multi-angle cooperative operation, a binary map of construction workers’ identification area is obtained as a mask using the method applied in foreground extraction with the redundant background of sub-images removed. The SURF feature extraction and matching are performed to improve the accuracy of matching. In the OpenCV visual library, function cv2.SURF() is utilized to create an instance of the SURF features and compute the SURF features of the image through its member function detectAndCompute(). After obtaining SURF features of images from two different angles, feature matching was performed by brute force method, which is realized by the instance member function knnMatch() of class cv2.BFMatcher, as shown in Figure 11. Through the above methods, the information obtained by multiple cameras is integrated to ensure the detection of construction workers with a wide coverage.

## 4. Safety Status Analysis

### 4.1. Definition of Hazardous Area

In construction sites, there are many possible dangerous areas because of the existing dangerous source. The defining mechanism of the dangerous area from identifying the dangerous source can be established. In construction safety accidents, the factors causing hazards are mainly classified into two categories. The first category refers to the energy factors and material factors that exist in the building safety system and may be released accidentally, such as potential energy, electromagnetic energy, thermal energy, mechanical energy, and other energy factors, as well as material factors such as inflammable and easy to explode substances, corrosive substances, toxic substances, and radioactive substances. The second category is the equipment, measures, containers, and other unsafe factors that cause the limitation or constraint of the first category of unsafe factors to fail or destroy. The occurrence of construction safety accidents is the result of the joint action of the above two types of factors, with the first type of factors as the prerequisite for the occurrence of accidents and the second type of factors as the necessary conditions. Common types of building injuries and corresponding hazard sources are shown in Table 1, through which the high frequency area of safety accidents on the construction site can be defined artificially and then the location information of the corresponding area is input into the program. When the program predicts that the personnel may enter the dangerous area, it outputs the warning information to the on-site safety manager and the corresponding workers to assist the safety manager in making management decisions.

### 4.2. Estimation of Workers’ Trajectory

For localization of the construction worker in the real coordinate3 system (world coordinate system $X_{W}Y_{W}Z_{W}$), the conversion relation between system $X_{W}Y_{W}Z_{W}$ and system uov is established. According to Formula (25), the coordinates of construction workers in the real world can be obtained.

(25) $$S\left[\begin{array}{c} X_{W}\\ Y_{W}\\ 1 \end{array}\right]=W_{3\times 3}^{-1}M_{3\times 3}^{-1}\left[\begin{array}{c} u\\ v\\ 1 \end{array}\right]$$

The factor *S* in Formula (25) is the depth information lost by the three-dimensional projection to the two-dimensional angle. To determine its value, a calibration board is set at *h* (*h* = 1 m), and the measured value of *S* is 0.085624526612. By connecting the positions of each construction worker at each moment, the corresponding movement trajectory of construction worker can be obtained to track the construction worker. The calibrated lens is used to shoot video clips to check the accuracy of the positioning method.

After identifying and locating the construction workers, Kalman filter is used to predict the workers’ movement. In the OpenCV visual library, the class cv.CreateKalman() is used to create the Kalman filter, which requires three input parameters, namely the dimension of the state space, the dimension of the measurement value (measure_param), and the dimension of the control vector (control_params). The member variable of the class cv.CreateKalman() (transition_matrix[]) is used to set the state transition matrix. In the case of setting the process noise covariance matrix, measuring noise covariance matrix, and initializing the posteriori error covariance matrix, the observation state can be predicted by applying the cv.KalmanPredict() method, and the observation state can be updated according to cv.KalmanCorrect() function.

### 4.3. Determination of Safety Status

According to Kalman filter, the movement position of construction workers can be predicted to determine whether the construction workers tend to enter the pre-set dangerous area. If there is a worker who will enter the dangerous area, they will be fed back to the on-site managers, to attract the attention and provide the basis for the managers to give safety management instructions.

**Figure 12:**
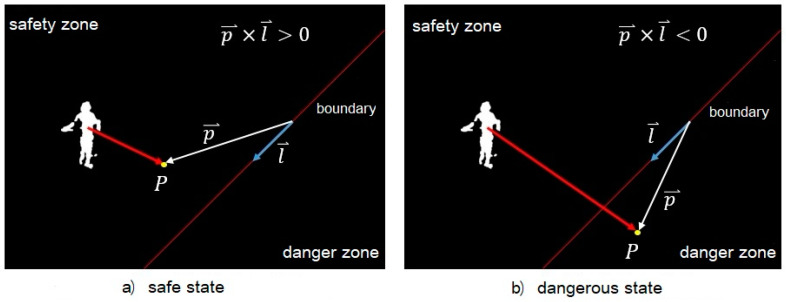
Method of danger determination.

After obtaining the movement trajectory of the construction workers, a discriminant method is used to control the issuing of the warning signal. As shown in Figure 12, the red line for dangerous zone boundary is input artificially. When the prediction point of a worker’s one-step trajectory is outside the danger zone, the cross product of the direction vector $\vec{l}$(counterclockwise in a straight line around the inside of the danger zone) of danger zone’s boundary and the prediction point vector $\vec{p}$ of the worker’s movement is positive. When the prediction point of the worker’s one-step trajectory is in the danger zone, the cross product of the direction vector $\vec{l}$ of the boundary of the danger zone and the prediction point vector $\vec{p}$ of the worker’s movement is negative.

## 5. Discussion

### 5.1. Worker Detection and Positioning

**Figure 13:**
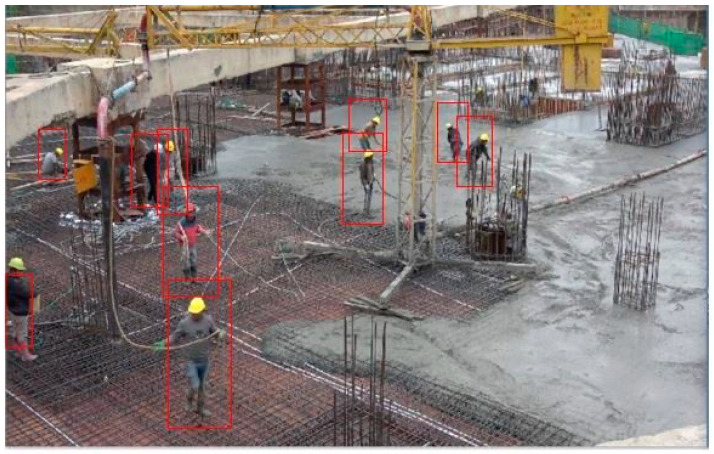
Worker detection.

**Figure 14:**
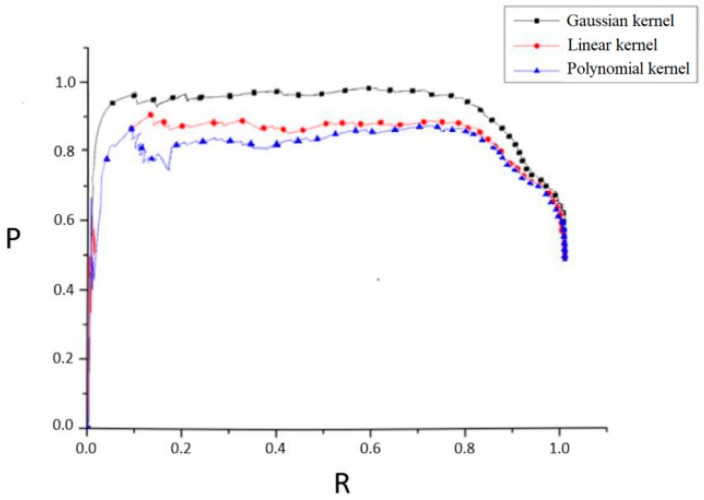
P-R curve of SVM classifier with different kernel function.

Based on the improved foreground extraction method, the worker detection and positioning model was established and tested. The effect of worker detection is shown in Figure 13, which demonstrates a considerable detection effect. To further improve the accuracy of worker detection, experiments were carried out to compare the classification effects under different kernels. The accuracy rate (P) and recall rate (R) of the models established with different kernels are shown in the Figure 14, according to which Gaussian kernel was finally selected as kernel function to train SVM classifier.

**Figure 15:**
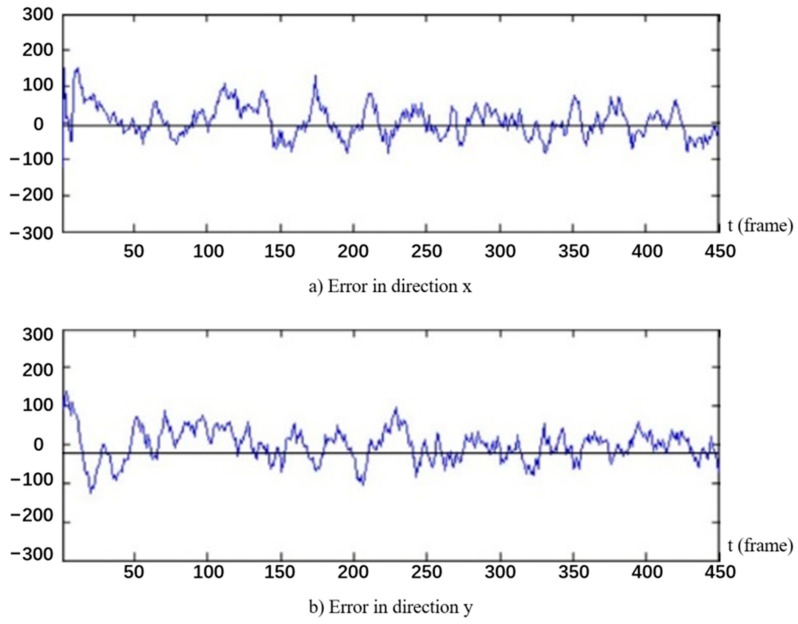
Positioning error.

Based on the worker detection model, the model of worker positioning in the real world was completed and tested. The result of positioning of construction workers is shown in Figure 15. As can be seen, the maximum value of positioning error is 1.531 m, while the mean value of positioning error in direction x is −0.109 m and the mean value of positioning error in direction y is −0.217 m. The results indicate that the proposed framework of worker identification and positioning can meet the requirements of prior warning.

### 5.2. Safety Status Monitoring

**Figure 16:**
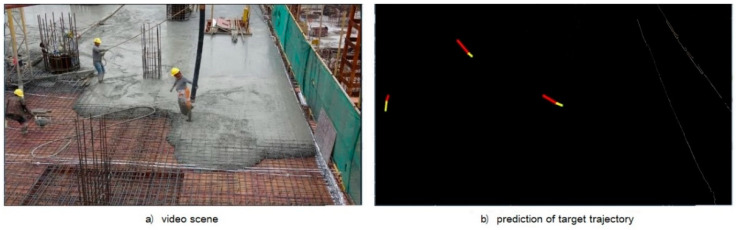
Field tracking effect.

**Figure 17:**
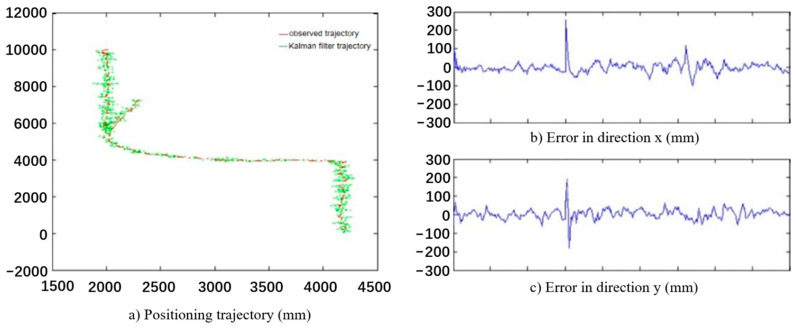
Result of tracking algorithm.

According to the definition of hazardous area in Section 4.1 and determination method of safety status in Section 4.3, the algorithm for worker’s safety status monitoring was also established and tested. The proposed algorithm was verified by the video scene shown in Figure 16a, and the experimental results are shown in Figure 16b, in which the yellow line segment represents the historical movement trajectory of worker, and the red line indicates the prediction of the worker movement trajectory. The test results are shown in Figure 17, which indicates that the error range of the Kalman filter tracking algorithm fluctuates within 300 mm, most of which are distributed within 100 mm.

Finally, five video clips were used to test the accuracy of the whole framework, and the average detection error of each video clip was calculated. The test results are shown in Table 2. The average error of each video clip is the weighted average of the ratio of the number of construction workers missed in each video frame to the actual number of workers. It can be seen from Table 2 that the average error of the algorithm is 9.444%, and the recognition accuracy is 90.666%.

### 5.3. Comparison of Computational Efficiency

In general, this paper presents an improved workers’ detection method with multi-angle information fusion, realizing a workers’ recognition accuracy of 90.666%. Meanwhile, workers’ movement trajectories are estimated with an error fluctuating within 300 mm, most of which are distributed within 100 mm, reaching a relatively accurate result of trajectory tracking and estimation. The determination and prediction of workers’ movement trajectories are implemented without any pre-positioned tags, solving the problem of setting them in advance [19]. Based on the detection and trajectories estimation of workers and the judgement of the positional relationship between workers and danger zone, an automatic analysis system for the safety status of on-site workers is established with its effect presented, overcoming the problem of manual operation [12,25,26].

Compared to previous studies [18,20], this study can realize accurate tracking with high identification accuracy. The improved algorithm with multi-angle information fusion helps to enhance the detection effect. It is also worth noting that although there is a trend of using deep-learning-based detection methods for on-site worker detection, the multi-angle fusion method proposed in this paper can still provide an idea to improve the detection effect, filling the knowledge gap proposed by Zhong et al. [24]. In addition, the framework proposed in this paper has a lower demand for computational resources in regarding the training of the detection model. As shown in Table 3, the deep learning model preciously developed by the authors [34] and the proposed method in this paper are compared by dealing with the same training set in the same equipment environment (a personal computer with 2.60 GHz CPU and 24 GB RAM) commonly seen on the construction site. The training time and response time during application are shown below. The method proposed in this paper can complete model training efficiently, with relatively short response time. If the equipment configuration is sufficient, the method proposed in this paper can realize considerable response speed and shorter training time as compared to deep-learning-based methods.

## 6. Conclusions

After reviewing the literature referring to the safety management of on-site workers, this paper proposed a method to make up for the knowledge gap on multi-angle detection of workers and implemented a pre-warning method for construction workers’ dangerous status. For the aspect of workers’ detection on the construction site, the improved GMM was adopted to extract the motion foreground, so as to reduce the influence on the algorithm caused by sudden changes in the construction site environment. Meanwhile, the HOG + SVM framework was utilized to complete the detection and classification of sub-images of moving objects, and finally the detection of workers on the construction site was implemented. According to the detection results of the proposed algorithm, it can be considered that the algorithm can accurately detect the construction workers in the video. It is worth mentioning that SURF algorithm was applied for information fusion of construction workers in multiple angles, which implements the detection of workers in multiple cameras. Finally, Kalman filter was applied to estimate the movement trajectory of workers on the construction site and determine the safety status. The location of construction workers on the construction site was tracked and predicted with results showing that the algorithm can track and predict workers’ moving position.

There are some drawbacks of this study that need further research. In the fusion of construction workers from multiple angles, the program is susceptible to the influence of camera resolution, and the detection effect of construction workers will be limited when they are far away from the camera. The presented study fails to provide a device that can offer an early warning signal. Therefore, in future research, investigating the use of substantial equipment (such as safety helmet of vibration) is still required to provide workers with an early warning.

## Figures and Tables

**Table 1:** Source of hazards.

Accident Types	Hazards’ Location	Energy Source
Fall from height	Site elevation difference, lifting appliance	Human body
Object strike	Solid falling, throwing, flying equipment, sites, operations	Object
Vehicle injury	Vehicle, traction equipment, ramp	Vehicle
Lifting injury	Crane, gantry crane, derrick	High-altitude heavy object
Mechanical injury	Mechanical driving device	Motion device or human body
Electric shock injury	Power supply, wire exposed	Electrified body
Fire injury	Storage of flammable material	Flame or smoke
Burning injury	Heat source device, self-heating object	High temperature substance
Poisoning injury	A device, container, or place for the production and storage of hazardous substances	Toxic substance
Explosion injury	Explosive material	Explosive
Collapse depression	Slopes, piles, buildings, structures	Soil mass

**Table 2:** Mean error of worker’s safety status monitoring.

Video Time (min)	Number of On-Site Workers	Specific Error	Mean Error
1	7	10.882%	9.444%
2	11	7.660%	
5	6	5.214%	
5	13	9.265%	
10	5	6.555%	
20	10	7.645%	

**Table 3:** Comparison of different methods’ computational efficiency.

Method	Training Time (h)	Response Time (s)
Our method	3.7	3.2
Deep learning	10.5	2.3

## Data Availability

Not applicable.
